# On the Complexity of Mechanisms and Consequences of Chromothripsis: An Update

**DOI:** 10.3389/fgene.2019.00393

**Published:** 2019-04-30

**Authors:** Alla S. Koltsova, Anna A. Pendina, Olga A. Efimova, Olga G. Chiryaeva, Tatyana V. Kuznetzova, Vladislav S. Baranov

**Affiliations:** ^1^D.O. Ott Research Institute of Obstetrics, Gynecology and Reproductology, Saint Petersburg, Russia; ^2^Department of Genetics and Biotechnology, Saint Petersburg State University, Saint Petersburg, Russia

**Keywords:** chromothripsis, complex chromosomal rearrangements, epigenetics, cancer, benign tumour, chromosome pulverisation, constitutional chromothripsis

## Abstract

In the present review, we focus on the phenomenon of chromothripsis, a new type of complex chromosomal rearrangements. We discuss the challenges of chromothripsis detection and its distinction from other chromoanagenesis events. Along with already known causes and mechanisms, we introduce aberrant epigenetic regulation as a possible pathway to chromothripsis. We address the issue of chromothripsis characteristics in cancers and benign tumours, as well as chromothripsis inheritance in cases of its occurrence in germ cells, zygotes and early embryos. Summarising the presented data on different phenotypic effect of chromothripsis, we assume that its consequences are most likely determined not by the chromosome shattering and reassembly themselves, but by the genome regions involved in the rearrangement.

## Introduction

Complex chromosomal rearrangements have been found since introduction of cytogenetic techniques. At present, due to development of new molecular-cytogenetic and molecular methods, the nature of CCRs became apparent making possible their classification.

The first documented CCR case was a translocation affecting three chromosomes in a child with mental retardation and associated dysmorphic features (Nuzzo et al., 1968). In 1970, a team of Lund University researchers discovered another translocation involving three, or possibly, four chromosomes and characterised it as a “complex translocation” and “complex rearrangement” (Fredga and Hall, 1970). Subsequently, complex chromosomal translocations were given the definition that currently extends to the term “CCRs”: complex chromosomal translocations involve more than a reciprocal exchange of segments between two chromosomes resulting in multiple derivative chromosomes (Pai et al., 1980). As molecular genetic techniques gained popularity, our understanding of the nature and origins of structural chromosomal abnormalities increased. As a result, the initial definition of CCRs is frequently updated in terms of the number of breakpoints and number of involved chromosomes. At present, CCRs are understood to be structural chromosomal abnormalities that arise as a result of three or more breakpoints in one or more chromosomes, with the exception of inter- and intrachromosomal insertions (Madan, 2013; McGowan-Jordan et al., 2016).

In January 2011, Stephens et al. (2011) published a paper on CCRs in chronic lymphocytic leukaemia. Using paired-end DNA sequencing, they revealed 42 rearrangements affecting chromosome 4 and several rearrangements affecting chromosomes 1, 12, and 15 in the tumour cells of one patient. The detected rearrangements were characterised not only by numerous breakpoints in a relatively short genome region but also by multiple deletions in the almost complete absence of duplications. Subsequently, when studying similar genome alterations in a small-cell lung cancer cell line (SCLC-21H), the authors observed the formation of double minutes from fragments of derivative chromosome 8 (Stephens et al., 2011). Notably, the rearranged chromosomes and the double minutes comprised material from only one of the homologous chromosomes, the other remaining intact. The authors suggested the term “chromothripsis” to describe this phenomenon (from the Greek “chromos” – “chromosome” – and “thripsis” – “shattering” into small fragments) (Stephens et al., 2011).

Importantly, apart from chromothripsis, over the last 7 years two more CCR types have been described: chromoanasynthesis and chromoplexy. The three types of aberrations are covered by the umbrella term “chromoanagenesis” (from the Greek “anagenesis” – “rebirth”), which indicates a structural chromosome reorganisation (Holland and Cleveland, 2012). It is believed, however, that chromothripsis differs from other chromoanagenesis phenomena by the mechanisms of its occurrence and the nature of genetic alterations (Poot, 2018).

## Chromothripsis and Other Types of Chromoanagenesis

**FIGURE 1:**
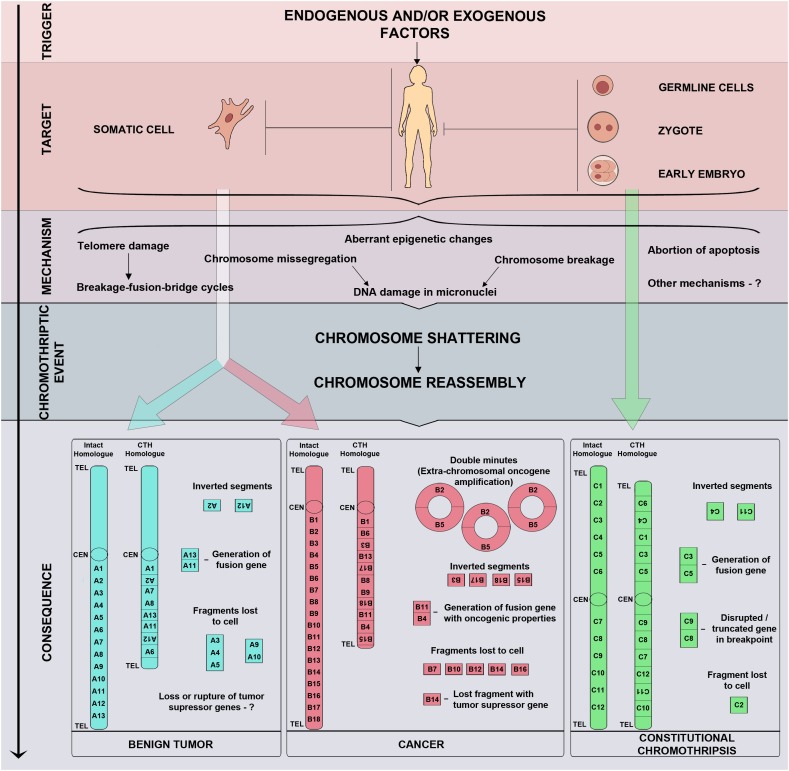
Triggers, mechanisms, and consequences of chromothripsis. Chromothripsis may arise in any cell, including somatic cells, germline cells, zygotes, and blastomeres of preimplantation embryos, thus, determining the fate of an affected organ or the whole organism. Chromothripsis is induced by exogenous and/or endogenous factors which trigger chromosome shattering and sequential reassembly of fragments through micronuclei formation, breakage-fusion-bridge cycles, aberrant epigenetic regulation, abortive apoptosis, and other yet unknown mechanisms.

The results of whole genome sequencing, followed by mapping reads against a reference genome, lead us to believe that chromothripsis is based on the process of chromosome shattering triggered by double-strand DNA breaks (Stephens et al., 2011). The repair of double-strand breaks in a cell may occur through either a homologous recombination or NHEJ (reviewed in Ceccaldi et al., 2016). NHEJ is believed to be the primary repair mechanism in chromothripsis cases (Stephens et al., 2011). Once the DNA has been repaired through NHEJ, the reassembled chromosome may have errors in the order and orientation of segments. Fragments that do not ligate together with a centromere may be lost during subsequent cell divisions resulting in deletions (Figure 1; MacKinnon and Campbell, 2013). When double-strand breaks occur in two or more chromosomes, chromosome fragments may fuse, forming derivative chromosomes.

In theory, such CCRs may result from either chromosome pulverisation or sequential, independent rearrangements. The Monte-Carlo simulation method, which includes repeated random sampling and is traditionally used in stochastic process research, has established that the chromosome pulverisation model, which implies an absence of duplications, more accurately matches the genome alterations observed in chromothripsis. These data have given rise to an assumption that chromothripsis is the result of a single catastrophic event (Stephens et al., 2011).

The discovery of chromothripsis in the tumour cells of patients with chronic lymphocytic leukaemia was followed by a description of constitutional chromosomal rearrangements that are comparable with chromothripsis by number of breakpoints and breakpoint clustering but have different copy-number profiles. Microarray results have revealed that the karyotype of 17 patients with various developmental problems featured not only deletions but also multiple duplications and triplications, which could not have arisen as a result of NHEJ (Liu et al., 2011). This enabled the authors to hypothesise that such copy number alterations may result from replication and repair errors caused by DNA microhomology (MMBIR, microhomology-mediated break-induced replication; MMIR, microhomology/microsatellite-induced replication) (Payen et al., 2008; Hastings et al., 2009). Since chromothripsis does not fully reflect the characteristics of the observed genome alterations, the authors suggested replacing the term “chromothripsis” with “chromoanasynthesis,” which stands for chromosome reconstitution or chromosome reassortment (Liu et al., 2011).

In their review article on the hypothetical mechanisms and consequences of chromoanagenesis, Holland and Cleveland (2012) contrast the terms “chromoanasynthesis” and “chromothripsis.” According to the authors, chromoanasynthesis and chromothripsis are two independent phenomena with different underlying mechanisms. However, multiple chromosomal aberrations, which are observed in both, are most likely the result of a single catastrophic event, and not a successive series of rearrangements.

By contrast, chromoplexy, the third example of chromoanagenesis, is the result of an accumulation of chromosome rearrangements. The term “chromoplexy” (from the Greek “pleko” – “to weave”) was introduced in 2013 to indicate complex rearrangements of prostate cancer genomes (Baca et al., 2013). To analyse the results of whole-genome sequencing and microarray-based comparative genomic hybridisation (aCGH), the researchers developed the ChainFinder algorithm, which identifies the chained rearrangements that resulted in the CCRs. They demonstrated that, in the majority of samples (50 out of 57), multiple deletions and translocations occurred successively, which is uncharacteristic of either chromothripsis or chromoanasynthesis. Chromoplexy is also characterised by fewer breakpoints and a larger number of rearranged chromosomes (up to eight) compared to chromothripsis (Baca et al., 2013). Importantly, the breakpoints are presumably localised in open chromatin regions (Berger et al., 2011). Therefore, the high transcription level of certain loci may serve as a chromoplexy trigger.

Recent study showed a novel potential mechanism of chromoanagenesis: DNA polymerase 𝜃-dependent alternative homologous end joining (Masset et al., 2016). Thus, chromoanagenesis may be induced by a variety of mechanisms that lead to CCRs. In contrast to chromoplexy and chromoanasynthesis, chromothripsis is characterised by a larger number of breakpoints and a random order and orientation of chromosome segments after reassembly. Chromothripsis features a high frequency of deletions in the almost complete absence of duplications in localised genome regions. However, chromothripsis identification among the multitude of CCRs is challenged by a lack of distinct limitations on the number of breakpoints and other features. The authors suggest six criteria to distinguish chromothripsis from other CCRs (Korbel and Campbell, 2013):

1. Clustering of breakpoints;
2. Oscillation of copy number states between one and two which is consistent with mono- or disomy;
3. A prevalence of regions with interspersed loss and retention of heterozygosity;
4. A prevalence of rearrangements affecting a single haplotype, i.e., one of two homologous chromosomes;
5. Randomness of DNA fragment joins and order, and;
6. Ability to “walk” the derivative chromosome by joining breakpoints.

The authors used statistical algorithms to justify some of the criteria, but they did not report the minimal number of breakpoints, and admitted the possibility of partial tri- and tetrasomies (Korbel and Campbell, 2013).

Initially, CCRs with over 50 breakpoints were classified as chromothripsis (Stephens et al., 2011). However, this criterion was not always fulfiled in subsequent works. In a number of cases, rearrangements with 20 (Molenaar et al., 2012), 10 (Northcott et al., 2012; Rausch et al., 2012), or fewer (Chiang et al., 2012) breakpoints were treated as chromothripsis. Kinsella et al. (2014) drew attention to this issue in 2014. Using statistical simulation, they demonstrated that chromothripsis-like rearrangements may result from sequential rearrangement. Importantly, these results do not debunk the traditional hypothesis of the origins of chromothripsis but only emphasise the need for further research.

## Methods of Chromothripsis Detection

It has been possible to describe the features of chromothripsis due to mate-pair sequencing and paired-end sequencing. These methods work for structural variant detection and CCRs, as well as genome assembly and *de novo* sequencing (Miller et al., 2010). In the case of a CCR, mate-pair and paired-end sequencing with subsequent verification by Sanger sequencing not only determine the precise localisation of breakpoints, but also gains data on nucleic acid sequences at breakpoint junctions (Gao and Smith, 2017). In spite of their high cost and challenging methodology, mate-pair sequencing and paired-end sequencing are widely used in chromothripsis studies.

Another efficient method of detecting and studying chromothripsis is microarray-based comparative genomic hybridisation (array CGH, aCGH), which is frequently referred to as “virtual karyotyping” or “chromosomal microarray analysis.” Copy number analysis allows detection of deletions, duplications, and other aberrations as well as identification of their precise genome localisation and size. The resolution of this method is sufficient to detect submicroscopic aberrations. For higher resolution and information capacity, aCGH is combined with a single nucleotide polymorphism (SNP) array (Keren, 2014). As a method, aCGH is not without considerable limitations: it cannot detect balanced structural chromosomal aberrations or determine the order and orientation of derivative chromosome segments (Balajee and Hande, 2018).

For detection and localisation of a specific DNA or RNA sequence on a chromosome or in a cell, fluorescence *in situ* hybridisation (FISH) is frequently used. In chromothripsis studies, various FISH techniques are used, each of them addressing specific aspects in the identification of the derivative chromosome structure. SKY and multicolour FISH (M-FISH), with the use of whole chromosome probes conjugated with different fluorochromes, enables identification of chromosomes involved in a rearrangement. The multicolour-banding FISH technique (MCB-FISH) is a segment-specific variant of chromosome banding that allows one to determine the structure of an aberrant chromosome (Balajee and Hande, 2018). To map breakpoints on the chromosomes, locus-specific probes with known cytogenetic localisation may be used for FISH. A combination of SKY and *in situ* hybridisation with fluorescent locus-specific probes is used to determine the precise structure not only of derivative chromosomes but also of double minutes (Stephens et al., 2011).

In patients with hereditary diseases, chromothripsis may be detected by a conventional karyotyping of metaphases from peripheral lymphocytes. This technique allows identification of numerical and structural chromosomal abnormalities including translocations and inversions, which are frequently observed in CCR cases. However, the complex nature of CCRs makes their interpretation by conventional karyotyping alone difficult. Therefore, to precisely determine the structure of rearrangements in chromothripsis, it is necessary to use a complex approach that includes classical chromosome banding, visualisation of the aberrations on metaphase chromosomes by FISH and molecular genetic techniques.

## Causes and Mechanisms of Chromothripsis

The first assumptions regarding the mechanisms of chromothripsis were made by Stephens et al. (2011). The authors argue that DNA junction sequences and their localisation in the genome attests to chromosome pulverisation during mitosis at the stage of their highest condensation, not at the interphase stage. Today, several presumed causes of chromothripsis are listed (Meyerson and Pellman, 2011; Forment et al., 2012; Jones and Jallepalli, 2012; Maher and Wilson, 2012).

### DNA Damage in Micronuclei

The most accepted hypothesis of chromothripsis occurrence is chromosome pulverisation in micronuclei. Chromosomes and their acentric fragments that lag during segregation in mitosis may be incorporated in a nuclear envelope outside of the main nucleus, which leads to the formation of micronuclei (Leibowitz et al., 2015). Certain features of the micronuclear envelope facilitate the access of cytoplasmic nucleases to the DNA (Géraud et al., 1989; Terradas et al., 2016). Micronuclei are characterised by abnormalities in chromatin condensation, which may lead to chromosome breaks (Terzoudi et al., 2015; Zhang et al., 2015). Experimental studies have shown the possibility of chromosome fragmentation and the formation of double minutes in micronuclei (Crasta et al., 2012; Hatch and Hetzer, 2015; Terradas et al., 2016). Using SKY, the authors determined that the majority of metaphases from cells with micronuclei feature multiple small fragments from one or two chromosomes (Crasta et al., 2012). The experiment on chromosome Y centromere inactivation also shed light on certain details of chromothripsis in micronuclei (Ly et al., 2017). The missegregated chromosome Y was included in a micronucleus and fragmented as a result of premature chromatin condensation. After the DNA breaks were repaired through NHEJ, the re-ligated chromosome Y showed typical characteristics of chromothripsis. It has been established that chromothripsis in micronuclei results from chromosome missegregation, their fragmentation, and the repair of breaks that occur during three cell cycles (Ly et al., 2017).

### Aborted Programmed Cell Death

The abortion of apoptosis is regarded as one of the causes of chromothripsis (Tubio and Estivill, 2011; Tang et al., 2012). The first data on the association of chromothripsis with mutations of *TP53*, which encodes p53 protein – the key apoptosis regulator – were obtained in 2012. Chromothripsis was detected in Sonic Hedgehog (SHH) medulloblastoma cells in a patient with hereditary Li-Fraumeni syndrome (a germline mutation of *TP53*) (Rausch et al., 2012). In acute and chronic lymphocytic leukaemia, TP53 mutations may co-occur with chromothripsis in tumour cells (Pei et al., 2012).

In 2015, the occurrence of chromothripsis in *TP53*^-/-^ cells after doxorubicin treatment on a cell-based model system was confirmed (Mardin et al., 2015). Observing a higher frequency of chromothripsis in hyperploid medulloblastomas, as compared to diploid ones, the authors established an association between cell hyperploidisation and chromothripsis. In this regard, it has been suggested that hyperploidisation may serve as a risk factor for chromothripsis (Mardin et al., 2015).

### Telomere Shortening and Formation of Dicentric Chromosomes

Highly localised rearrangements in chromothripsis can also be explained by breakage-fusion-bridge cycles in dicentric chromosomes, which arise from DNA damage or telomere fusion caused, in turn, by telomere shortening or loss (Stephens et al., 2011; Sorzano et al., 2013). When dicentric chromosomes segregate during mitosis, chromatin bridges are formed and undergo subsequent rupturing (McClintock, 1939). Having induced the formation of an envelope with an aberrant structure, the chromatin bridge is destroyed by cytoplasmic 3′-exonuclease TREX1 (Maciejowski et al., 2015; Maciejowski and de Lange, 2017). This may result not only in multiple losses and inversions of chromosome segments but also in the formation of double minutes. Breakage-fusion-bridge cycles may co-occur with fragment amplification, as demonstrated on regions of chromosome 21 (iAMP21) in a dicentric chromosome formed as a result of a Robertsonian translocation of chromosomes 15 and 21 (Li et al., 2014). The risk of iAMP21 acute lymphoblastic leukaemia in carriers of rob (15;21) is assessed to be ∼2700 times higher than in the population (Li et al., 2014). The presence of breakage-fusion-bridge cycles in cells with chromothripsis has also been demonstrated in studies of cancer genome alterations (Nones et al., 2014; Maciejowski et al., 2015; Ernst et al., 2016). These cycles, however, may be a part of neochromosome evolution and therefore, considering that neochromosomes arise through chromothripsis, may be the consequence, not the cause, of the phenomenon (Garsed et al., 2014).

### Chromosome Pulverisation Caused by Exogenous Factors

Chromosome pulverisation is an extreme example of DNA fragmentation. Multiple double-strand breaks in the DNA may result from exposure to a range of DNA-damaging agents including drugs, therapeutic or environmental ionising radiation, oxidative stress and virus infections.

Despite the initial suggestion that ionising radiation may induce chromothripsis, experimental proof was not obtained until several years later. In their experiments, Morishita et al. (2016) used a focused vertical microbeam system designed to irradiate a spot within the nuclei – the Single Particle Irradiation system to Cell (SPICE) – on oral squamous-cell carcinoma cells. The authors then established irradiated monoclonal sublines from them and analysed genome abnormalities using SKY and SNP array. One of the 46 monoclonal sublines showed chromothripsis-like complex chromosomal alterations with 14 breakpoints. The involvement of 10 chromosomes in the rearrangement is explained by the exposure of the interphase nuclei to a powerful particle beam. The authors presume that cell irradiation during mitosis may induce chromosome missegregation and, as a result, lead to micronuclei formation (Morishita et al., 2016).

Another potential cause of chromothripsis is chromosome pulverisation in viral infections. A connexion has been established between chromosome pulverisation and fragmentation and infection of cell cultures with measles, herpes zoster, herpes simplex, and adenovirus types 4, 12, and 18 (Benyesh-Melnick et al., 1964; Nichols et al., 1965; O’Neill and Miles, 1970; Peat and Stanley, 1986). In addition, herpes simplex may induce cell polyploidisation, which is also a risk factor for chromothripsis (Chenet-Monte et al., 1986; Mardin et al., 2015). Tumour cells infected with the Epstein-Barr virus have an increased level of both transmissible and unstable chromosomal abnormalities (dicentric chromosomes, chromatid fragments, ring chromosomes, double minutes, satellite associations of acrocentric chromosomes, and chromatin breaks) (Kamranvar et al., 2007). However, only one of the studies (Schütze et al., 2016) confirms the association of chromothripsis with viral infections. In human foreskin keratinocytes culture infected with human papillomavirus, chromothripsis-like complex chromosomal alterations within chromosome 8 occurred after passage 30, were detected at passage 40, and resulted in a gain of *MYC*. Concurrently, immortalisation of the cell line *in vitro* with non-transformed phenotype was observed (Schütze et al., 2016).

While the listed causes of chromothripsis appear to be the most likely, it is necessary to consider other possible contributing factors such as mutations in DNA repair genes or abnormal chromatin condensation.

### Aberrant Epigenetic Patterns as a Cause of Chromosome Damage

Chromothripsis is characterised by a high frequency of deletions, translocations and inversions (Stephens et al., 2011). These chromosomal aberrations result from multiple double-strand breaks (DSBs) possibly occurring during M or G1 phase. DSBs are most probably repaired by error-prone NHEJ or microhomology-mediated end joining (MMEJ) mechanisms (Jones and Jallepalli, 2012). In most cases, very short or no microhomology in the chromothripsis breakpoint junctions can be found (Stephens et al., 2011; Chiang et al., 2012; Kloosterman et al., 2012; Malhotra et al., 2013; Weckselblatt et al., 2015; Aristidou et al., 2018; Slamova et al., 2018). However, in a few cases of CC, DSBs were found in high-copy repeats (Nazaryan et al., 2014; Nazaryan-Petersen et al., 2016).

The chromatin conformation is of importance for occurrence of spontaneous DSBs. The transition from closed to open chromatin, which is necessary for transcription, makes DNA vulnerable to damage (Kuo, 1981; Falk et al., 2008; Meschini et al., 2015). Chromatin looping facilitates DNA cleavage by nucleases, including endogenous ones originating from transposable elements (Maniotis et al., 2005). In the study on the events involved in the occurrence of stably segregating CC, DNA cleavage by catalytically active L1-endonuclease and translocations between distally located DNA regions were explained by Alu-mediated chromatin looping (Nazaryan-Petersen et al., 2016). Enhanced activation of transposable elements is associated with a response to environmental change and as well as with syndromes caused by *MeCP2* (methyl-CpG binding protein 2; involved in transcription regulation) and *ATM* (ataxia telangiectasia, mutated; involved in DNA repair machinery) mutations (Bundo et al., 2014).

A key role in the regulation of chromatin structure belongs to epigenetic mechanisms: DNA methylation, histone variants, and non-coding RNAs (Geiman and Robertson, 2002; Li, 2014). Both tumorigenesis and cell differentiation including embryonic and germline cells are characterised by extensive epigenetic changes (Yamaguchi et al., 2013; Efimova et al., 2015, 2017, 2018; Avgustinova and Benitah, 2016; Atlasi and Stunnenberg, 2017; Pendina et al., 2018). Epigenetic machinery provides fast response to environmental change through gene-specific and/or genome-wide alterations of DNA methylation with subsequent change in expression patterns of genes coding proteins and regulatory RNAs (Wang et al., 2017; West, 2017). Abnormal DNA methylation also may compromise genome integrity. *In vivo* increase of chromosome aberrations has been documented in tissues with reduced global DNA methylation caused by ionising radiation (Lee et al., 2015), oxidative stress (Tunc and Tremellen, 2009), or deregulated DNMTs (Gaudet et al., 2003). In blood cells of ICF patients having *DNMT3a* mutation, hypomethylation of 1q, 9q, 16q heterochromatin regions is associated with abnormal chromatin looping, telomeric associations, anaphase bridges, lagging chromosomes, chromosome breakage and micronuclei formation (Gisselsson et al., 2005). In addition, hypomethylation of pericentromeric heterochromatin may trouble kinetochore orientation and spindle attachment, resulting in chromosome missegregation and micronuclei formation (Luzhna et al., 2013). Thus, aberrant DNA methylation contributes to abnormal chromatin compaction and, as a consequence, to DNA damage.

The involvement of epigenetic mechanisms in the pathway between damaging agents and genome integrity has been established in the studies of the radiation-induced bystander effect. The bystander effect is a phenomenon whereby irradiated cells communicate damage to non-irradiated nearby bystander cells, thus destabilising their genome and contributing to carcinogenesis (Koturbash et al., 2007). In rodents, localised X-ray exposure modifies expression of DNA methyltransferases and 5-methylcytosine-binding protein MeCP2 genes leading to global hypomethylation both in irradiated and non-irradiated tissues *in vivo* (Koturbash et al., 2006, 2007; Tamminga et al., 2008). DNA damage in non-irradiated bystander tissues is associated with induction of apoptosis (Koturbash et al., 2008; Kovalchuk et al., 2010; Cordelli et al., 2012). Recent advances in bystander effect aetiology assumed that communication between irradiated and non-irradiated cells involves numerous microRNAs (Xu et al., 2015; Yuan et al., 2016; Cai et al., 2017). In addition to microRNAs, cell-free chromatin released from radiation-induced dying cells is involved in extensive chromosome instability of bystander cells (Kirolikar et al., 2018).

Summarising the abovementioned issues, it could be assumed that activation of the cellular mechanisms involved in the chromothripsis formation by exogenous and/or endogenous insult is epigenetically mediated. However, lack of experimental evidence directly linking disruption of epigenetic regulation to the initiation of chromothripsis substantiates further studies in this field.

## Chromothripsis and Neoplasia

In 2015, ChromothripsisDB^1^ database was created (Yang et al., 2016) to categorise cases of chromothripsis in human and model organisms by disease, research method, and criteria that enabled the authors to classify the observed chromosomal abnormalities as chromothripsis. As of March 2018, the database counted 500 chromothripsis cases across 46 cancers. The authors of ChromothripsisDB update it on a regular basis and standardise the information on all the rearrangements that are treated as chromothripsis (Cai, 2018). At present, ChromothripsisDB is the most informative source of information for accessing and comparing the results of chromothripsis studies.

### Chromothripsis in Cancers

Chromothripsis is typical for 2–3% of cancer types (Stephens et al., 2011). As of today, chromothripsis has been observed in blood cancers, central nervous system cancers, soft tissue tumours, and carcinomas (Rode et al., 2016).

The frequency of chromothripsis varies across tumour entities (Table 1). Chromothripsis occurs most frequently in bone cancers – osteosarcoma and chordoma (Stephens et al., 2011). It is associated with advanced stages of the disease and poor clinical outcomes (Forment et al., 2012). At times, chromothripsis is coupled with additional mutations in tumour cells, for instance, *IDH* mutations (Cohen et al., 2015). In addition, the occurrence of chromothripsis in cancers is considerably higher in patients with inherited genetic disorders that are linked to cell-cycle and DNA repair gene mutations: Li-Fraumeni and Louis-Bar syndromes (Rausch et al., 2012; Ratnaparkhe et al., 2017). The risk of chromothripsis also varies across different genome regions: chromosomes 17, 8, 12, and 11 are the most likely to be involved in such rearrangements. As it appears, the highest frequency of chromothripsis in chromosome 17 is predetermined by the presence of the *TP53* gene in its short arm (Cai et al., 2014).

**Table 1:** Types of cancer with the highest occurrence of chromothripsis.

References	Cancer type	Cases with chromothripsis/ total number of cases	Chromothripsis frequency
Rausch et al., 2012	SHH medulloblastoma with *mut TP53*	10/10	100%
Rausch et al., 2012	SHH medulloblastoma with *wt TP53*	0/22	0%
Northcott et al., 2012; Rausch et al., 2012	Medulloblastoma, all subgroups	13/98; 139/1087	13%
Li et al., 2014	Acute lymphoblastic leukaemia with iAMP21	8/9	89%
Morrison et al., 2014	Invasive bladder carcinoma	81/150	60%
Zemanova et al., 2014	Myelodysplastic syndrome with CCR	77/157	49%
Rausch et al., 2012	Acute myeloid leukaemia with *mut TP53*	8/17	47%
Rausch et al., 2012	Acute myeloid leukaemia with *wt TP53*	1/91	1%
Przybytkowski et al., 2014	High-risk breast cancer	12/29	41%
Malhotra et al., 2013	Grade IV glioma (glioblastoma)	7/18	39%
Cohen et al., 2015	Grade IV glioma with *mut IDH*	9/24	37%
Cohen et al., 2015	Grade II–III glioma	5/45	11%
Malhotra et al., 2013	Lung adenocarcinoma	2/6	33%
Stephens et al., 2011	Osteosarcoma	3/9	33%
Nones et al., 2014	Esophageal adenocarcinoma	40/123	32%

*iAMP, amplification of a chromosome 21 region; IDH, isocitrate dehydrogenase; SHH, Sonic Hedgehog.*

### Chromothripsis in Benign Tumours

Chromothripsis does not occur exclusively in malignant tumours; cases of chromothripsis have been observed in benign tumours as well. The year 2013 brought the first descriptions of chromothripsis in uterine leiomyoma (also called uterine fibroid) cells – a benign tumour of the uterine myometrium, which is characterised by a high frequency of chromosomal abnormalities. By various estimates, chromothripsis occurs in 13–42% of uterine fibroids (Mehine et al., 2013; Holzmann et al., 2014; Mehine et al., 2014).

Unlike malignant tumours, chromothripsis in uterine fibroid cells is characterised by fewer breakpoints (20 or more) and a larger number of affected chromosomes (up to four) (Figure 1). Such aberrations are normally observed in uterine fibroids without fibroid-specific *MED12* (mediator complex subunit 12) and *FH* (fumarate hydratase) mutations. They do not feature *TP53* mutations or histological signs of malignancy (Mehine et al., 2013; Holzmann et al., 2014; Mehine et al., 2014; Pendina et al., 2017). Furthermore, chromothripsis with large deletions (from 43 to 13,647 kbp) has been observed in non-cultured sample of uterine fibroid which demonstrated normal karyotype in culture conditions (Holzmann et al., 2014). This could be associated with a lower proliferative potential of tumour cells with chromothripsis *in vitro*. However, a case of unbalanced chromothripsis has been observed in both the cultured and non-cultured fibroid cells (Pendina et al., 2017). It is likely that the ability of fibroid cells with chromothripsis to proliferate *in vitro* is determined not so much by the size of deletions and number of breaks as by the genomic loci involved in rearrangement. It should be noted, however, that the absence of malignisation signs in fibroids with chromothripsis by no means implies that their growth and malignant potential does not require thorough study.

## Constitutional Chromothripsis as a Consequence of Genome Damage in Germ Cells and Preimplantation Embryos

Chromothripsis may also be a constitutional karyotype abnormality caused by chromosome damage in germline cells or preimplantation embryos. Cases of CC are extremely rare and usually coincide with congenital malformations or reproductive failure in the patient (Table 2; Kloosterman et al., 2011;de Pagter et al., 2015). In the virtually complete absence of any genetic imbalance, CC may co-occur with breakage of multiple genes or changes in their expression (Table 2; van Heesch et al., 2014; de Pagter et al., 2015; Bertelsen et al., 2016; Middelkamp et al., 2017). CC may include structural chromosomal abnormalities associated with genetic disorders (Table 2; Fontana et al., 2014; Genesio et al., 2015; Kurtas et al., 2018). In this case, the patient displays symptoms of an inherited disease. However, certain non-specific phenotypical features complicate the diagnosis and prognosis of the clinical outcome of the CC (Table 2).

**Table 2:** Clinical outcomes of constitutional chromothripsis.

References	Chromosome regions involved in chromothripsis	Chromothripsis detection method	Imbalance (size, copy number alterations)	Affected genes	Phenotype of a carrier(s)
Bertelsen et al., 2016; Nazaryan-Petersen et al., 2016	3q22.3-q23 5q23.1	Conventional cytogenetics, mate-pair sequencing	Four deletions (2–110 kb)	Truncated genes: *PPP2R3A*, *CLDN18*, *A4GNT*, *DBR1*, *HSD17B4*, *ATR* Fusion genes: *CLDN18-HSD17B4*, *HSD17B4-DBR1* Deleted genes: *DZIP1L*	No apparent association with a disorder
Anderson et al., 2016	13q33.1-q33.3 Xp11.22-p21.3 Xq21.31-q22.1	Conventional cytogenetics, FISH, aCGH	10 deletions (327 kb – 8 Mb): a total 4.4 Mb of chr. 13 material and 28.1 Mb of chr. X material	Deleted genes: Chr. 13 – *ERCC5*, *SLC10A2* Chr. X – *IL1RAPL1*, *DMD*, *GK*, *NROB1*, *CYBB*, *OTC*, *RPGR*, *TSPAN7*, *XK*, *ATP6AP2*, *BCOR*, *CASK*, *CFP*, *KDM6A*, *MAOA*, *NDP*, *NYX*, *RBM10*, *RP2*, *SYN1*, *UBA1*, *USP9X*, *ZNF81*, *BMP15*, *CACNA1F*, *CLCN5*, *FOXP3*, *HSD17B10*, *IQSEC2*, *KDM5C*, *PHF8*, *FGD1*, *HUWE1*, *HSD17B10*, *DIAPH2*, *SRPX2*	Developmental delay and dysmorphism
Weckselblatt et al., 2015	1q21 4q31 7p14.3 15q22	Conventional cytogenetics, FISH, targeted sequencing	530-kb deletion of chr. 1 material; 4,2-Mb duplication of chr. 7 material	No disrupted genes by the breakpoints	Developmental delay, autism, intellectual disability, and/or congenital anomalies
	3q25-q26 8q23 9p22-p24 11p14.1 3q21.1	Conventional cytogenetics, FISH, WGS	Mb-sized deletions of chr. 8 and 9 material; a total of 99 bp deleted of other chromosomes material	Disrupted genes by the breakpoints: *PTPRD*, *SH3GL2*	Developmental delay, autism, intellectual disability, and/or congenital anomalies
	2q32-qter 3q13 7q21.11-q22.1 10q21.3 11q14.1	Conventional cytogenetics, FISH, WGS	800-kb deletion of chr. 7 material, 2.2-Mb deletion of chr. 11 material; in addition, there are 55 total bp deleted at breakpoint junctions on other chromosomes	Disrupted genes by the breakpoints: *GRM3*, *KPNA1*, *DLG2*, *CACNA2D1*, *GULP1*, *COL5A2*, *KCNH7*, *PCLO*, *TRRAP*	Developmental delay, autism, intellectual disability, and/or congenital anomalies
Nazaryan et al., 2014	2p16.1-p22.1 5p14.2-p15.2 7p21.3-q31.1	Conventional cytogenetics, FISH, mate-pair sequencing	No copy number alterations	Truncated genes: *CDH12*, *DGKB*, *FOXP2*	Global developmental and psychomotor delay, severe speech disorder
Gamba et al., 2015	1p36.33-p35.3	Conventional cytogenetics, aCGH	Five deletions: 0.83, 0.94, 1.4, 1.7, 3.7 Mb 1 duplication: 5.9 Mb	No data	Multiple congenital malformations presenting some features overlapping the 1p36 deletion phenotype
Gu et al., 2013	5p13.3-p15.33 7p22 7q32 11q23 21q21	Conventional cytogenetics, FISH, aCGH	No copy number alterations	No data	Phenotypically normal
	5p13.3-p15.33 11q23	Conventional cytogenetics, FISH, aCGH	Three deletions: 2.89, 0.56, and 3.21 Mb	Deleted genes: *LOC340094*, *ADAMTS16*, *KIAA0947*, *FLJ33360*, *MED10*, *UBE2QL1*, *LOC255167*, *NSUN2*, *SRD5A1*, *PAPD7*, *MIR4278*	Phenotypically normal
	5p13.3-5p15.33	Conventional cytogenetics, FISH, aCGH	∼26.22-Mb deletion	No data	Developmental delay, dysmorphic and autistic features
Kloosterman et al., 2011	1p32.3 4q24 10q21.1	Conventional cytogenetics, SNP array, mate-pair sequencing	Small deletions and duplications (<50 bp)	Disrupted gene: *PCDH15*	Severe psychomotor retardation, speech delay, hypertelorism and kyphoscoliosis
Slamova et al., 2018	1q23-q25 6q15-q24 14q13? 18p11.2-p11.3 18q11.2	Conventional cytogenetics, FISH, aCGH, mate-pair sequencing	Two deletions: 0.7 and 2.5 Mb	Deleted genes: *DNM3*, *PIGC*, *C1ORF105*, *SUCO*, *NMBR*, *VTA1*, *ADGRG6*, *HIVEP2*, *AIG1*, *ADAT2*, *PEX3*, *FUCA2*, *PHACTR2*, *LTV1*, *ZC2HC1B*, *PLAGL1*, *SF3B5*, *STX11*, *UTRN*, *PAX9* Disrupted genes by the breakpoints: *FILIP1*, *PHIP*, *HMGN3*, *AK097143*, *GAREM*	Developmental and growth delay
Wang et al., 2015	19p13.13-p13.2 19p12 19q12 19q13.11-q13.12	Conventional cytogenetics, FISH, aCGH	Four duplications: 4.3, 0.98, 1.12, and 5.13 Mb	No data	Subtle dysmorphic features
Macera et al., 2015	3p24.3 5q14 7q35 9p23 18p11.31 18q21.31	Conventional cytogenetics, FISH, SNP array, NGS	No loss or gain of chromosomal material at any of the breakpoints	Disrupted genes by the breakpoints: *CNTN6*, *TBC1D5*, *CNTNAP2*, *PTPRD*, *L3MBTL4*, *LOC1001304840*, *WDR7*	Bilateral ventriculomegaly (13 and 15 mm), colpocephaly, with partial agenesis of the corpus callosum, and an absent left kidney and small right kidney
Kurtas et al., 2018	22q13.1-q13.3	Conventional cytogenetics, FISH, aCGH, WGS, WES	Two duplications: 2.4 Mb, 148 kb 1 deletion: 8.4 Mb	Disrupted genes by the breakpoints: *EP300*, *NFAM1*, *MYO18B*, *GTPBP1*	Phelan-McDermid syndrome
Genesio et al., 2015	9p21-q31	Conventional cytogenetics, FISH, aCGH	Two deletions: 176.56 kb, 7.44 Mb	Deleted genes: *RORB*, *TRPM6*, *NMRK1*, *OSTF1*, *GNAQ*, and the critical region of the 9q21.13 deletion syndrome	Platelet disorder and thyroid dysfunction in addition to the classical neurobehavioral phenotype of the 9q21.13 microdeletion syndrome
Del Rey et al., 2016	2q34-q37.3	Conventional cytogenetics, FISH, HR-CGH, MLPA	Deletion: 2.58 Mb duplication of 2q34q37.2	Deleted genes: *K1F1A*, *PASK*, *HDLBP*, *FARP2*	Multiple congenital disorders and intellectual disability
Fontana et al., 2014	1q41 1q43 9p24.3 21q22.12	Conventional cytogenetics, aCGH	Four deletions: 5.23, 1.33, 0.15871, and 0.826 Mb	Deleted genes: *SMYD2*, *PTPN14*, *CENPF*, *KCNK2*, *KCTD3*, *USH2A*, *ESRRG*, *SPATA17*, *RRP15*, *TGFB2*, *CHRM3*, *KANK1*, *RCAN1*, *CLIC6*, *RUNX1*	Loeys–Dietz syndrome, type 4; borderline mental impairment
Kurtas et al., 2019	3q22.3-q26.2	Conventional cytogenetics, FISH, aCGH, paired-end sequencing	Deletion: 6.8 kb	Disrupted genes by the breakpoints: *ROPN1B*, *NAALADL2*, *TF*	Healthy
	3q22.3-q26.2	Conventional cytogenetics, FISH, aCGH, paired-end sequencing	Duplication: 10 Mb deletion: 5 Mb	Disrupted genes by the breakpoints: *ROPN1B*, *NAALADL2*, *TF*	Multiple phenotypic abnormalities and psychomotor delay
	chr. 6 14q31.3	Conventional cytogenetics, FISH, aCGH, paired-end sequencing	Two deletions: 5.3 and 3.7 kb	Disrupted genes by the breakpoints: *OPRM*, *RNGTT*	Healthy
	chr. 6 14q31.3	Conventional cytogenetics, FISH, aCGH, paired-end sequencing	Deletion: 1 Mb	Disrupted genes by the breakpoints: *OPRM*, *RNGTT*	Healthy
	15q15.1 6p21.3-p25.1 6q14.2 6q21-q22.31 7q32.3	Conventional cytogenetics, FISH, SNP-CGH array, paired-end sequencing	Deletion: 6 kb	Disrupted genes by the breakpoints: *CASC5*, *RPF2*, *CHCHD3*, *CLVS2*	Healthy
	15q15.1 6p21.3-p25.1 6q14.2 6q21-q22.31 7q32.3	Conventional cytogenetics, FISH, SNP-CGH array, paired-end sequencing	Four deletions up to 100 bp 6-bp microduplication	Disrupted genes by the breakpoints: *CASC5*, *RPF2*, *CHCHD3*, *CLVS2*. One parental breakpoint junction is absent	Developmental and speech delay, dysmorphic features

*aCGH, array comparative genomic hybridisation; FISH, fluorescent in situ hybridisation; HR-CGH, high resolution comparative genomic hybridisation; MLPA, multiplex ligation-dependent probe amplification; NGS, next generation sequencing; SNP array, single nucleotide polymorphism array; WGS, whole genome sequencing; WES, whole exome sequencing.*

Constitutional chromothripsis carriers may transmit the rearrangement to the offspring either stably or with *de novo* events (Gu et al., 2013; Weckselblatt et al., 2015; Bertelsen et al., 2016; Nazaryan-Petersen et al., 2016; Kurtas et al., 2019). Whereas the majority of *de novo* CC cases result from chromosomal aberrations arising from male gametogenesis (Pellestor et al., 2014), chromothripsis is inherited primarily from the mother (Table 3). To all appearances, it is determined by differences in DNA repair capacity and specific features of spermatogenesis and oogenesis.

**Table 3:** Chromothripsis inheritance.

References	Maternal inheritance, cases	Paternal inheritance, cases	*De novo* chromothripsis, cases
Kloosterman et al., 2011	–	–	1 (pat)
Kloosterman et al., 2012	1	–	7 (4/7 – pat; 3/7 – n/d)
Gu et al., 2013	1	–	–
Nazaryan et al., 2014	–	–	1
Fontana et al., 2014	–	–	1
Wang et al., 2015	–	–	1
Genesio et al., 2015	–	–	1
de Pagter et al., 2015	3	–	–
Gamba et al., 2015	–	–	1
Weckselblatt et al., 2015	1	1	1 (pat)
Del Rey et al., 2016	–	–	1
Anderson et al., 2016	–	–	1
Bertelsen et al., 2016	3	1	–
Collins et al., 2017	–	–	2
Kurtas et al., 2018	–	–	1
Kurtas et al., 2019	2	1	–
Total:	10	3	19

*De novo chromothripsis cases include information on the parental origin of the rearranged chromosomes (if known). pat, paternal chromosomes; n/d, no data about derivative chromosome origin.*

Chromothripsis may arise during mitotic and meiotic divisions of spermatogenic cells as well as during spermiogenesis (round spermatid differentiation in spermatogonia) (Pellestor and Gatinois, 2018). Considering that spermatogonia undergo a succession of mitotic divisions, the replication stress may lead to errors during mitosis. Meiotic recombination may also feature double-strand break repair errors (Pellestor and Gatinois, 2018). The DNA breaks in spermatids that occur at the stage of histone-to-protamine transition during spermiogenesis can only be repaired through NHEJ because of the haploid chromosome number in cells at this stage (Gunes et al., 2015). In rodent male germ cells, scaffold/matrix-attached and differentially packaged chromatin regions are highly sensitive to endogenous nucleases, and, thus, to damage (Arpanahi et al., 2009; Grégoire et al., 2013). Accumulation of DNA strand breaks may be also caused by the epigenetically mediated bystander effect in non-irradiated whole testis tissue (Tamminga et al., 2008). This phenomenon is also involved in the production of delayed DNA damage in mouse elongated spermatids due to upregulation of proapoptotic genes 21–33 days later after spermatogonia exposure to X-rays (Cordelli et al., 2012). However, the apoptotic elimination of spermatogenic cells with DNA damage may be aborted before completion (the so-called abortive apoptosis or anoikis), allowing such cells to continue to differentiate and participate in fertilisation (Tubio and Estivill, 2011; Tang et al., 2012). In addition, there are some evidence of aberrant DNA methylation and tissue-specific accumulation of chromosome aberrations in unexposed progeny of cranially irradiated rodents (Koturbash et al., 2006; Tamminga et al., 2008). These data indicate an epigenetic link between DNA damaging agents and occurrence of chromosome aberrations both in unexposed parental germline and offspring’s somatic cells.

In contrast to male germ cells, oocytes may repair breaks through both homologous recombination and NHEJ (Marchetti et al., 2007). Consequently, chromothripsis during oogenesis appears to be less likely than during spermatogenesis. Aberrations in chromosome segregation and premature chromatid separation may cause chromosomal rearrangements during female gametogenesis (Pellestor and Gatinois, 2018). In addition, the DNA repair capacity of an oocyte is the crucial factor of zygote viability, because the repair of maternal and paternal chromosome damage after fertilisation and prior to embryo genome activation occurs through DNA repair factors accumulated in the oocyte cytoplasm.

*De novo* CC may also be induced by DNA damage during early embryogenesis. Preimplantation embryos typically demonstrate micronuclei formation, blastomere fragmentation, and abnormal mitosis at the cleavage stage (Chavez et al., 2012). This could be a consequence of imperfect repair in germ cells or DNA damage in embryo. In addition, asynchronous pronuclear development and resulting under-replication of the paternal DNA may induce chromosome pulverisation in a zygote (Eichenlaub-Ritter et al., 1995).

Importantly, CCRs are hardly ever detected during conventional karyotyping of chorion cells in a miscarriage, which is conducted starting from 4 to 5 weeks of gestation (i.e., after embryo implantation) (Pendina et al., 2014; Massalska et al., 2017; Soler et al., 2017; Pylyp et al., 2018). As of today, the literature describes only one case of CC in an embryo with multiple malformations (Macera et al., 2015). Apparently, most embryos with CCRs, including chromothripsis, are eliminated at the implantation stage. Despite the wide use of preimplantation genetic testing, the actual frequency and the specific mechanisms of chromothripsis occurrence in gametes and embryos at early stages of development are yet to be determined.

Constitutional chromothripsis is generally characterised by fewer chromosome breaks and almost complete absence of deletions in comparison with malignant tumours (Figure 1; Kloosterman and Cuppen, 2013). A number of studies treat CCR cases with duplications of chromosome regions as chromothripsis (Table 2; Gamba et al., 2015; Wang et al., 2015; Del Rey et al., 2016; Kurtas et al., 2018). It is yet to be established, however, whether such genetic abnormalities in patients are cases of true chromothripsis or variations of other CCRs.

## Concluding Remarks

As is the case with any recently discovered phenomenon, the concept of chromothripsis is ambiguous. In our opinion, the most comprehensive definition of chromothripsis has been suggested by Ly and Cleveland: “Chromothripsis is a catastrophic event in which one or a few chromosomes are shattered and stitched back together in random order, producing a derivative chromosome with complex rearrangements within a few cell cycles” (Ly and Cleveland, 2017). Considering that chromothripsis is a highly complex genomic aberration, its reliable detection necessitates the use of a comprehensive approach, combining molecular genetic, molecular cytogenetic, and cytogenetic methods.

Chromothripsis was first detected in chronic lymphocytic leukaemia. To date, it is most frequently found in cancers, even though there are registered cases of chromothripsis both in benign tumours and as constitutional chromosomal abnormality. Both somatic and CC feature multiple rearrangements of one or more chromosomes with a random order and orientation of reassembled fragments, as well as alteration of regions with loss and retention of heterozygosity. However, these aberrations are less pronounced in CC, which normally has fewer breaks and shorter chromosome regions with copy number alterations or a complete absence of such.

The causes and mechanisms underlying chromothripsis remain a subject for discussion. The most probable are telomere damage, exposure to ionising radiation, and viral infections. Along with these already known causes and mechanisms, we suggest aberrant epigenetic regulation as a possible pathway to chromothripsis. The above-mentioned factors may directly destruct chromosomes or activate cell mechanisms associated with chromothripsis. To clearly understand chromothripsis mechanisms, it is necessary to develop models of chromosome pulverisation in micronuclei, reversible apoptosis, and dicentric chromosome breaks.

As of today, it is not clear whether somatic chromothripsis is the cause of tumours or a consequence of pathological processes in tumour cells. Considering that cases of chromothripsis are observed in both malignant and benign tumours, as well as in the karyotype of healthy individuals, it cannot be unambiguously associated with poor clinical outcomes. Apparently, what matters most for neoplasia pathogenesis and a chromothripsis carrier’s phenotype are the genome regions involved in the rearrangement, their localisation, and the size of deleted or amplified fragments – not the presence of chromothripsis itself.

Regardless of the fact that chromothripsis was discovered over 7 years ago, we are still facing challenges in its differentiation from other multiple chromosomal rearrangements and in the understanding of its causes, mechanisms, and consequences – all of which requires further in-depth research.

## Author Contributions

AK, AP, OE, OC, TK, and VB contributed to the conception, writing, and checking of the manuscript for important intellectual content.

## Conflict of Interest Statement

The authors declare that the research was conducted in the absence of any commercial or financial relationships that could be construed as a potential conflict of interest.

## Footnotes

1 http://cgma.scu.edu.cn/ChromothripsisDB

## Abbreviations

aCGH: array comparative genomic hybridisation
CC: constitutional chromothripsis
CCR: complex chromosomal rearrangement
DSB: DNA double-strand break
FISH: fluorescent *in situ* hybridisation
NHEJ: non-homologous end joining
SKY: spectral karyotyping
SNP array: single nucleotide polymorphism array
